# Association of Chronic Pain with Motor Symptom Severity in Parkinson’s Disease: An Exploratory Cross-Sectional Analysis

**DOI:** 10.3390/life15020268

**Published:** 2025-02-11

**Authors:** Niels Pacheco-Barrios, Vivian D. B. Gagliardi, Roberta R. Grudtner, Iloba Gabriel Njokanma, Ben Illigens, John D. Rolston, Felipe Fregni, Kevin Pacheco-Barrios

**Affiliations:** 1 Carrera de Medicina Humana, Universidad Científica del Sur, Lima 15067, Peru; niels_pacheco@hms.harvard.edu; 2 Department of Neurosurgery, Brigham and Women’s Hospital, Harvard Medical School, Boston, MA 02130, USA; jrolston@bwh.harvard.edu; 3 Hospital Israelita Albert Einstein, São Paulo 05652-900, Brazil; vivian.gagliardi-2022@ppcr.org; 4 São Pedro Psychiatric Hospital, Secretaria Estadual da Saúde Rio Grande do Sul, Porto Alegre 90450-190, Brazil; roberta.grudtner-2019@ppcr.org; 5 Lagos State University Teaching Hospital, Lagos 100271, Nigeria; iloba.njokanma-2020@ppcr.org; 6 Department of Neurology, Beth Israel Deaconess Medical Center, Harvard Medical School, Boston, MA 02130, USA; ben.illigens@bi.harvard.edu; 7 Neuromodulation Center and Center for Clinical Research Learning, Spaulding Rehabilitation Hospital and Massachusetts General Hospital, Harvard Medical School, Boston, MA 02130, USA; fregni.felipe@mgh.harvard.edu; 8 Unidad de Investigación para la Generación y Síntesis de Evidencias en Salud, Universidad San Ignacio de Loyola, Lima 15023, Peru

**Keywords:** chronic pain, Parkinson’s disease, motor function

## Abstract

Background: Parkinson’s disease (PD) is a neurodegenerative disorder characterized by motor symptoms like bradykinesia, tremor, rigidity, and postural instability. Additionally, PD severely impacts physical abilities and independence. Chronic pain, affecting 67.6% of PD patients, varies in form and presentation, and it is often underdiagnosed. Objectives: This study investigated the association between chronic pain and motor symptom severity in PD patients. Methods: This analysis used data from a cross-sectional study on 52 Parkinson’s disease (PD) patients conducted at Jena University Hospital, Germany. The dataset, available on Dryad, included demographics; clinical reports; and assessments of coping strategies, quality of life, and pain. Descriptive statistics, a bivariate analysis, and an ordinal logistic regression model were executed to explore the association between pain and motor symptom severity (MSS). A direct acyclic graph was used to represent the relationship between variables and identify potential confounders, and an outcomes definition sensitivity analysis was used to assess the impact of using pain intensity as an outcome. The E-value was calculated to evaluate the strength of association needed by an unmeasured confounder to nullify the observed association. Results: A total of 50 Parkinson’s disease (PD) patients were included, with 64% being male, with an average age of 76.1 years. The sample included 20 patients without pain and 30 with chronic pain. The bivariate analysis did not identify significant differences in disease duration, cognitive function, and non-motor symptoms between pain and no-pain groups. However, significant differences (*p*-value < 0.05) emerged in motor symptom severity, coping strategies, and several SF-36 domains (Physical and Social Functioning, Role Functioning, Energy/Fatigue, Pain, General Health, and Health Change). The ordinal logistic regression showed a substantial association between chronic pain and MSS: patients with chronic pain had 3.52 times higher odds (95% CI: 1.40–8.84, effect size d ≈ 0.70, *p* = 0.02) of low to medium MSS and 5.44 times higher odds (95% CI: 2.03–14.60, effect size d ≈ 0.94, *p* = 0.01) of medium to severe MSS, indicating a dose–response relationship. Additionally, male patients had increased odds of higher MSS (OR 4.63, 95% CI: 1.15–18.58, effect size d ≈ 0.85, *p* = 0.03). Conclusions: Chronic pain is strongly associated with MSS in PD patients, with a more pronounced effect as MSS progresses from medium to severe, supporting a dose–response relationship. Effect sizes suggest a robust association, emphasizing the need for pain assessment in managing motor symptoms in PD.

## 1. Introduction

Parkinson’s disease (PD) is a neurodegenerative disorder that affects more than 8.5 million individuals [1], and it is projected to affect more than 9.3 million by 2030 [2]. Motor symptoms are the hallmark of this disease: bradykinesia, resting tremor, rigidity, and postural instability. These symptoms arise from the progressive loss of dopaminergic neurons in the substantia nigra, a critical region in the brain’s motor circuitry [3]. As it progresses, PD imposes substantial physical limitations, leading to a decrease in independence and daily functioning [4].

The progression of motor symptom severity (MSS) plays a pivotal role in the clinical course of PD. Elevated MSS levels not only shape therapeutic decisions but also exacerbate the disease burden on patients, affecting both their quality of life and ability to manage daily tasks [5,6,7]. Efforts to identify prognostic factors influencing MSS progression have included the age of disease onset, duration since diagnosis, and the presence of cognitive decline or dementia [8]. However, pain, a prevalent and impactful symptom experienced by approximately 67.6% of PD patients, remains underexamined as a factor in MSS progression [8,9].

While motor symptoms are the most visible manifestation of Parkinson’s disease (PD), pain is an equally critical yet often underappreciated aspect that significantly affects patient quality of life. Moreover, pain is a very frequent symptom, affecting approximately 67.6% of PD patients [9]. This pain can manifest in various forms, ranging from musculoskeletal discomfort to neuropathic or dystonic pain, each with distinct characteristics and implications. Moreover, it has been reported that PD patients have a lower pain threshold than healthy controls [10]. As a result, some studies have studied the association of pain and MSS [11,12,13], showing contradicting results. Notably, two studies [11,12] reported that higher pain scores are associated with worse motor performance, while another study observed the opposite relationship [13].

PD patients can experience various types of pain, each with distinct characteristics, contributing to a complex pain profile that often becomes chronic [14]. Chronic pain in PD is defined as pain lasting for more than three months, impacting daily functioning and quality of life [15]. Pain in PD patients can be categorized as musculoskeletal, neuropathic, dystonic, akathitic, and central pain [16]. The most common type, musculoskeletal, stems from rigidity and muscle stiffness and causes discomfort in joints, neck, and back. Neuropathic pain results from nerve damage or altered pain processing, manifesting as burning or tingling sensations, often in the limbs or face. Dystonic pain arises from involuntary muscle contractions, leading to painful cramps or spasms, particularly during “off” medication periods when PD symptoms worsen. Additionally, akathitic pain, linked to inner restlessness, drives patients to move frequently to relieve discomfort, typically seen in advanced PD stages. Finally, central or primary pain is believed to result from changes in the brain’s pain pathways, causing diffuse, hard-to-localize pain that may resist typical treatments. Recognizing these pain types is essential for comprehensive pain management, as chronic pain significantly impacts mobility, independence, emotional well-being, and overall quality of life in PD patients.

The exact mechanisms linking pain with PD are not fully understood, and pain often remains underdiagnosed and undertreated in this population [17]. For instance, it is often challenging to distinguish how much of chronic pain in PD patients is attributable to the disease itself versus the typical aging process, as the average age of individuals with PD is over 60 years [18]. In addition, the reported prevalence of chronic pain in PD varies widely, ranging from about 24% to 83% [19,20,21,22], with the latest estimate being at 20% [23].

Understanding the nature of pain in PD is complex, as it may be directly related to the disease pathology, a consequence of motor symptoms, or even a side effect of medication [24]. This multifaceted nature of pain in PD patients necessitates a deeper investigation, particularly in how it is associated with the severity of motor symptoms, an area that has received limited focus in existing research. Because of this, our study aims to evaluate the association of pain and MSS in PD patients.

## 2. Methods

### 2.1. Data Source, Study Design, Setting, and Participants

This secondary data analysis used a dataset from a cross-sectional study published in an open data publishing platform, the journal *Data in Brief* [25]. The dataset is shared through Dryad, allowing for the direct download of the dataset stored in Microsoft Excel [26]. This dataset was selected as it provides comprehensive, granular information on pain-coping strategies and health-related quality of life, specifically tailored to this unique patient population. It was the only open dataset identified, to our knowledge, that offers detailed and relevant pain-related data in PD patients, making it particularly valuable for addressing our research question. This dataset included 52 patients with PD by consecutive sampling between May 2019 and July 2019 to receive PD-specialized multimodal treatment in the neurological ward at the Jena University Hospital, Jena, Germany. The ethics committee of the Jena University Hospital (4572-10/15) approved the study. Informed consent in accordance with the Declaration of Helsinki was obtained from all participants. The assessments were collected before the beginning of multimodal complex therapy. The inpatient admission criteria were an increase in fluctuations, an increase in off phases, evaluation for deep brain stimulation, and worsening of gait and freezing. Subjects with the Montreal Cognitive Assessment (MOCA) score above 21 points were included in the study.

### 2.2. Variables and Data Measurements

The dataset contains demographic variables such as age, sex, disease duration, and housing situation, as well as clinical data reports, including pain, cognition, depressive symptoms, motor and non-motor symptoms, and stage of functional PD disabilities. Coping strategies and health-related quality of life were measured using the Coping Strategy Questionnaire (CSQ) and the SF-36 score, respectively. Moreover, the Short Form 36 (SF-36) item 21 (evaluating pain during the past 4 weeks) and Item 22 (How much did pain interfere with your normal work, including work outside the home and housework, in the past four weeks?) were reported. In addition, the Montreal Cognitive Assessment (MOCA) was used for cognitive evaluation; the Movement Disorder Society-sponsored review of the Unified Parkinson’s Disease Rating Scale III (MDS UPDRS III) and the Revised Non-Motor Symptoms Questionnaire (NMS-Quest) were used for the assessment of motor and non-motor symptoms; the Beck Depression Inventory was used to assesses the severity of depressive symptomatology; and the Hoehn and Yahr Scale was applied for the staging of PD patients.

The exposure, pain, was defined using the Item 21 from SF-36 subscale (“How severe was your physical pain in the past four weeks?”), a variable composed of six ordinal categories. We converted the item 21 into a categorical variable, defining “no pain” if the participant scored one, two, or three and “presence of pain” if they scored four, five, or six in this item. In addition, the variable pain intensity was defined using the ordinal scale of the item 21, with 0 = none, 1 = very mild, 2 = mild, 3 = moderate, 4 = severe, and 5 = very severe.

The primary outcome, motor symptom severity (MSS), was defined as a categorical variable from the MDS-UPDRS Part III, with three categories: low, medium, and high. For the categorization, we used previously validated cut-off points [27], defining the score from 0 to 35 as low, 36–46 as medium, and >46 as high severity.

### 2.3. Bias and Statistical Analysis

Statistical analysis was performed using Stata/BE 18.0 for Windows (64-bit x86-64), with a *p*-value of <0.05 indicating statistical significance. First, we summarized categorical variables using frequencies and continuous variables using central tendency and dispersion measurements. Visual inspection and a Shapiro–Wilk test were used to evaluate normality for the continuous variables. Means with standard deviations (SD) and medians with interquartile range (IQR) were used for normal and non-normal distributions, respectively. Second, a bivariate analysis was performed using chi2 for ordinal/categorical variables, the *t*-test, or the Mann–Whitney U test for continuous variables. An ordinal logistic regression model was executed to evaluate the association between pain and MSS, computing the odds ratios (OR), two-sided 95% confidence intervals, and *p*-values.

A sample size calculation was conducted to ensure adequate power for detecting an association between pain and motor symptom severity (MSS). Based on previous literature, we assumed a prevalence of chronic pain in Parkinson’s disease patients of approximately 20% [9,23]. Using Fisher’s exact test for two independent groups, with a significance level (α) of 0.05 and a power (1 − β) of 0.80, we determined that a minimum total sample size of 24 participants would be sufficient for our primary analysis. To increase statistical power to 0.90, a sample size of 32 participants would be needed. The sample size calculations were performed a priori to ensure that the study was adequately powered to detect a meaningful association and to address the potential for type II errors in the analysis.

A direct acyclic graph was used to identify potential confounders, effect modifiers, and colliders in our dataset. Moreover, an outcome definition sensitivity analysis was performed to evaluate the effect of using pain intensity as an outcome. To perform this, we changed the definition of the exposure from pain presence to pain intensity, both described in the previous section. Finally, the E-value was calculated using the estimates of the ordinal logistic regression to “define the minimum strength of association that an unmeasured confounder would need to have with both the treatment and the outcome to fully explain away a specific treatment-outcome association” [28]. The E-value was calculated using the web application available online [29]. The E-value represents the minimum strength of association that an unmeasured confounder would need to have with both the exposure (pain) and the outcome (MSS) to fully explain away the observed association. For instance, an E-value of 2 would mean that an unmeasured confounder would need to have an odds ratio of at least 2 with both pain and MSS simultaneously to nullify the observed association.

## 3. Results

### 3.1. Participant Demographics and Descriptive Analysis

The study sample comprised 50 patients, including 20 patients without pain and 30 patients with pain. Among them, 32 (64%) were male participants, aged 76.1 ± 6.5 years (range, 50 to >85 years). Thirty-five patients were not living alone, while 15 were living alone. Moreover, 24 patients had more than eight years of disease duration, while 26 had less than eight years of disease duration. In addition, 41 patients have stage three or more in the Hoehn and Yahr stage classification, as well as 17 patients who had a score between 21–23 in the MOCA scale. Only five patients obtained >20 in the Beck Depression inventory. Additionally, 48 patients experienced at least more than two non-motor symptoms. Furthermore, 26 patients had low MSS, 15 had medium MSS, and 9 had high MSS. Table 1 summarizes the demographic data of the participants.

### 3.2. Pain Effects in the Clinical Profile of PD Patients

There were no statistically significant differences between groups (no pain vs. pain) regarding disease duration and Hoehn and Yahr stage. Also, we did not find any statistically significant differences between MOCA assessments, BDI-II, and the Revised Nonmotor Symptoms Questionnaire.

There was a significant difference between groups regarding motor symptom severity MDS UPDRS III (no pain group: low severity 16/20, medium severity 2/20, high severity 2/20; pain group: low severity 10/30, medium severity 13/30, 7/30; *p* = 0.005), and also in two questions from the Coping Strategies Questionnaire: catastrophizing (no pain group mean 26.1 (SD 16.5) vs. pain group 41.5 (SD 17.4), *p* = 0.0068) and increasing activity level (no pain group 48.2 (18.6) vs. pain group 38.1 (18.6), *p* = 0.049).

Also, in the SF-36 form, there were statistically significant differences in the questions: physical functioning (mean, SD: 53.8 (23.5) vs. 29.2 (21.3), *p* < 0.001), social functioning (physical) (mean (SD): 30.0 (35.9) vs. 10.0 (22.4), *p* = 0.01), role functioning (emotional) (mean (SD): 61.7 (45.0) vs. 35.6 (41.9), *p* = 0.03), energy/fatigue (mean (SD): 52.8 (15.3) vs. 39.5 (13.4), *p* < 0.01), pain (mean (SD): 68.9 (19.8) vs. 31.1 (16.5), *p* < 0.01), general health (mean (SD): 48.0 (11.4) vs. 33.2 (14.2), *p* < 0.01), and health change (mean (SD): 40.0 (20.5) vs. 24; 2 (23.2), *p* < 0.01). Table 1 shows the detailed clinical and demographic characteristics of the patients in this dataset.

### 3.3. Association of Pain and Motor Symptom Severity

In the univariate analysis of PD pain and motor symptom severity (Table 2), there was 1.32 times (95% CI [1.31–2.41]) higher odds to shift from mild to medium MSS if pain was present. In addition, there were 2.95 times more odds (95% CI [1.65–4.26]) to shift from medium to severe MSS in patients with pain.

The multivariate logistic regression analysis included MSS, pain presence, age, disease duration, and Hoehn and Yahr state. The resulting main-effects model (Table 3) indicated pain and sex as significant explanatory variables. The estimates showed 3.52 times (95% CI [0.38–6.67]) higher odds to shift from mild to medium MSS if pain was present. Moreover, the estimate indicated 5.44 times (95% CI [2.03–8.86]) higher odds to shift from medium to severe MSS if pain was present. In addition, being a male was associated with 4.63 times higher odds of developing more severe motor symptoms (95% CI [1.07–17.66]). We did not observe any significant associations between disease duration and the Hoehn and Yahr state (Table 3). We included other confounder variables from the direct acyclic graph (Supplementary Figure S1) as potential confounders; however, none of them was retained in the final model due to no change in more than 10% of the regression coefficient or significant independent predictor.

### 3.4. Sensitivity Analysis

The sensitivity analysis of the multivariate logistic regression models (Table 4), using pain as an ordinal variable instead of a binary variable, showed similar results, presenting pain and sex as significant explanatory variables. The estimates showed 5.83 times (95% CI [2.11–9.55]) higher odds to shift from mild to medium MSS if pain was present. Moreover, the estimate indicated 5.44 times (95% CI [3.68–11.75] higher odds to shift from medium to severe MSS if pain was present.

**Figure 1:**
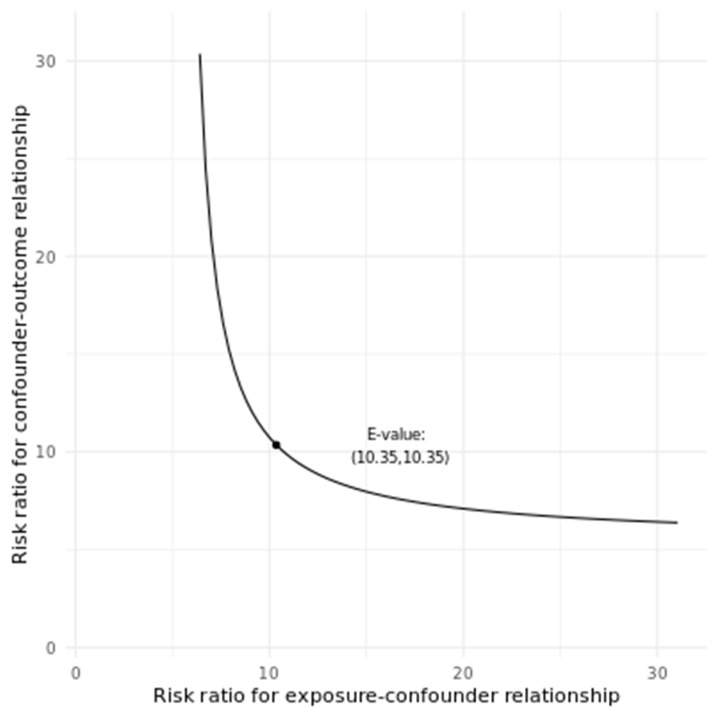
E-value calculation for the sensitivity analysis model. Performed in the web-based application available online [29].

In addition, with an observed OR of 5.44 95% CI [2.03–8.86] (pain presence associated with medium to severe MSS), an unmeasured confounder that was associated with both the outcome and the exposure by a OR of 10.35-fold each, above and beyond the measured confounders, could explain away the estimate, but weaker joint confounder associations could not (Figure 1). Furthermore, to move the confidence interval to include the null, an unmeasured confounder that was associated with the outcome and the exposure by an OR of 3.48-fold each could do so, but weaker joint confounder association could not.

## 4. Discussion

Our study demonstrates a significant association between pain presence and motor symptom severity (MSS) in Parkinson’s disease (PD) patients, suggesting that pain may serve as an indicator of worsening motor function. Multivariate logistic regression analysis showed that patients with pain had 3.52-fold increased odds of progressing from mild to moderate MSS and a 5.44-fold increase in odds of progressing from moderate to severe MSS, indicating a dose–response relationship. Clinically, these findings suggest that PD patients who experience pain may have a higher risk of accelerated motor decline, which could impact their daily functioning and quality of life more rapidly than those without pain. Furthermore, male patients were found to have higher odds of experiencing more severe motor symptoms, indicating potential sex-based differences in disease progression. Sensitivity analysis confirmed the robustness of these findings, with an E-value of 10.35 indicating strong resistance to unmeasured confounding.

Patients with pain may face increased difficulty with mobility, balance, and other motor tasks, potentially leading to greater reliance on assistance and a heightened need for supportive interventions [30]. This highlights the importance of regular pain assessment in PD patients, as pain could serve as an early indicator of motor deterioration, prompting healthcare providers to consider early interventions to manage both pain and motor symptoms, potentially slowing the progression of disability in affected patients.

Some studies have evaluated the association of pain and severity in Parkinson’s disease. For instance, a cross-sectional study conducted in 117 PD’s patients found pain is associated with motor symptoms [12], with an adjusted OR of 5.7 (95% CI, 2 to 16.5; *p* = 0.001) and a positive correlation of pain severity and MSS. In addition, another study also found an association of pain with Hoehn and Yahr staging (OR, 1.9; *p* = 0.04) and motor complications (OR, 4.7; *p* = 0.04) [11]. Another example [13] conducted correlation analyses to evaluate the relationship between pain and motor symptoms, finding similar results.

On the other hand, a study conducted by Li in 2021 [31] did not find an association between MSS and pain. This difference can be explained by the different types of scales used for pain measurement, the number of patients included, and the treatment received. However, the authors did not report the association estimate of pain and MDS-UPDRS part III as well as the model construction in detail, increasing the risk of bias and the possible effect of measured and unmeasured confounders.

Among all the previous studies, there was high heterogeneity in the sample sizes and the measurement of the exposures and outcomes. Sample sizes ranged from 38 to 180 patients. In addition, the measurement of pain was conducted using the NMS scale [11], yes–no questionaries [12], the King’s Parkinson’s Disease Pain Scale (KPPS), and the Brief Pain Inventory (BPI) [13]. These factors could have contributed to the heterogeneous results.

Moreover, PD is a complex and diverse neurodegenerative disorder, with motor symptoms that may arise from different underlying pathological mechanisms [32,33]. This heterogeneity extends to the experience of non-motor symptoms, such as pain, which may be influenced by various underlying neural circuits and disease processes. The relationship between these PD mechanism subtypes and pain has not been thoroughly explored, and this lack of investigation into PD’s heterogeneity could partially explain the inconsistent findings in the available literature. Understanding the diverse mechanisms underlying pain in PD could shed light on the variability in patient experiences and help identify more targeted therapeutic strategies.

Several theories have been proposed as the link of pain and motor symptoms in PD. Degeneration of nerve terminals in Aδ and C fibers has been shown to occur in PD patients, due to the deposition of phosphorylated alpha-synuclein in cutaneous sensory and autonomic nerves [34,35,36,37]. Moreover, depression, another frequent comorbidity that affects 38% of PD patients [38], has been shown as an important factor for pain presence in PD [39,40].

Furthermore, central pain processing has also been proposed to be altered in patients with PD due to the degeneration of both dopaminergic and non-dopaminergic pathways. However, the contribution of atypical top-down (central) processing and enhanced anticipation of pain has not been fully explored [41]. Additionally, the role of the subthalamic nucleus (STN) has caught great attention in explaining pain due to the nociceptive network involving this nucleus [42]. Another connection involved in the STN nociceptive network is the cerebellum, specifically the posterolateral portions (crus I and crus II), possibly contributing to the motor symptoms [43]. Some exploratory analyses have observed abnormalities of this network in PD patients [44,45]. However, it is still unclear if the STN is the most important factor when explaining pain in PD.

A better understanding of the molecular pathophysiology and the circuitry involved in pain and PDs could lead to improved pharmacological interventions or deep brain stimulation (DBS) targeting approaches to reduce pain among PD patients or even to reduce the progression of motor symptoms. For instance, analysis derived from a clinical trial evaluating the early application of STN-DBS in Parkinson’s disease has shown promising outcomes. Specifically, the odds of experiencing worse rest tremor for participants receiving early STN-DBS combined with optimal drug therapy (ODT) were only 0.21 times those of participants receiving early ODT alone (*p* < 0.001, OR 0.21, 95% CI 0.09 to 0.45) [46]. In the connectivity analysis of these patients, DBS modulation of fiber tracks originating from the cerebellum was linked to slower motor progression [47].

Our findings on the association between pain and motor symptom severity (MSS) in PD may provide a basis for future research exploring the broader implications of pain on patients’ quality of life, including aspects of social functioning and holistic well-being. Given the growing emphasis on holistic approaches in PD care, studies investigating the interplay between pain, social relationships, and communication could provide valuable insights. For instance, Takahashi et al. [48] highlighted the significance of social relationships in enhancing the quality of life in PD patients, suggesting that interventions that address pain could indirectly benefit social well-being. Moreover, Chen et al. [49] examined the relationship between neuropsychiatric features and daily activities, showing that these elements substantially affect social functioning in PD. These insights emphasize the importance of comprehensive approaches that consider both physical and social dimensions of PD care. Future studies might expand on our findings by exploring how managing pain could potentially mitigate the decline in social functioning and quality of life, especially in patients with progressive MSS.

Our study has some limitations. First, it is an exploratory analysis from a cross-sectional study, so it is not possible to establish a temporal relationship between pain and motor symptom severity. Second, the small sample size available prevented us from executing a more complex regression model due to the risk of overfitting. Third, the measurement of pain can be controversial, as our pain exposure comes from the SF-36 scale. Finally, medication status was not available in the dataset, converting this variable into a possible confounder. Further prospective studies may analyze and confirm our study findings, as well as evaluate such confounders using the appropriate PD-specific assessment tools to enhance the robustness and applicability of the research.

## 5. Conclusions

Our study reveals a significant association between chronic pain and motor symptom severity (MSS) in Parkinson’s disease (PD), especially in the progression from medium to severe stages, suggesting a dose–response relationship. Future research should enhance exposure and outcome measurement with PD-specific scales, account for the heterogeneity of PD motor symptom pathological mechanisms, and assess the temporal dynamics of pain and MSS.

## Figures and Tables

**Table 1:** Demographic characteristics of the patients in this dataset.

Variable	No Pain (n = 20)	Pain (n = 30)	Overall (n = 50)	*p*-Value
**Age (years)**				0.54
50–59	0 (0%)	1 (3%)	1 (2%)	
60–64	0 (0%)	2 (7%)	2 (4%)	
65–69	2 (10%)	5 (17%)	7 (14%)	
70–74	5 (25%)	7 (23%)	12 (24%)	
75–79	5 (25%)	9 (30%)	14 (28%)	
80–84	7 (35%)	6 (20%)	13 (26%)	
>85	1 (5%)	0 (0%)	1 (2%)	
**Sex**				
Male	15 (75%)	17 (57%)	32 (64%)	0.19
Female	5 (25%)	13 (43%)	18 (36%)	
**Housing situation**				1.00
Lives alone	6 (30%)	9 (30%)	15 (30%)	
Not alone	14 (70%)	21 (70%)	35 (70%)	
**Disease duration (years)**				0.29
1–3	4 (20%)	4 (13%)	8 (16%)	
4–7	10 (50%)	8 (27%)	18 (36%)	
8–11	2 (10%)	7 (23%)	9 (18%)	
12–15	2 (10%)	8 (27%)	10 (20%)	
>16	2 (10%)	3 (10%)	5 (10%)	
**Hoehn and Yahr stage**				0.66
1	1 (5%)	1 (3.3%)	2 (4%)	
2	4 (20%)	3 (10%)	7 (14%)	
3	13 (65%)	20 (66.7%)	33 (66%)	
4	2 (10%)	4 (13.3%)	6 (12%)	
5	0 (0%)	2 (6.7%)	2 (4%)	
**Montreal Cognitive Assessment (MOCA)**				0.35
21–23	8 (40%)	9 (30%)	17 (34%)	
24–27	9 (45%)	11 (37%)	20 (40%)	
28–30	3 (15%)	10 (33%)	13 (26%)	
**Beck Depression Inventory II**				0.73
0–2	0 (0%)	2 (7%)	2 (4%)	
3–4	3 (15%)	2 (7%)	5 (10%)	
5–6	2 (10%)	2 (7%)	4 (8%)	
7–8	8 (40%)	7 (23%)	15 (30%)	
9–10	1 (5%)	2 (7%)	3 (6%)	
11–12	1 (5%)	4 (13%)	5 (10%)	
15–16	2 (10%)	3 (10%)	5 (10%)	
17–19	2 (10%)	4 (13%)	6 (12%)	
>20	1 (5%)	4 (13%)	5 (10%)	
**Revised Nonmotor Symptoms Questionnaire**				0.106
0–2	2 (10%)	0 (0%)	2 (4%)	
3–5	4 (20%)	1 (3.3%)	5 (10%)	
6–8	3 (15%)	7 (23.3%)	10 (20%)	
9–11	4 (20%)	5 (16.7%)	9 (18%)	
12–14	1 (5%)	8 (26.7%)	9 (18%)	
15–17	5 (25%)	6 (20%)	11 (22%)	
>18	1 (5%)	3 (10%)	4 (8%)	
**Movement Disorder Society-Sponsored revision of the Unified Parkinson’s Disease Rating Scale III**				0.005
Low severity	16 (80%)	10 (33%)	26 (52%)	
Medium severity	2 (10%)	13 (43%)	15 (30%)	
High severity	2 (10%)	7 (23%)	9 (18%)	
**Coping Strategy Questionnaire (CSQ)**				
Diverting attention, mean (SD)	37.2 (18.9)	41.8 (19.5)	39.9 (19.2)	0.36
Reinterpreting pain sensations, mean (SD)	26.2 (15.5)	20.8 (16.3)	22.9 (16)	0.17
Coping self-statements, mean (SD)	55.0 (17.2)	51.2 (17.6)	52.7 (17.3)	0.53
Ignoring pain sensations, mean (SD)	46.8 (22.1)	38.0 (22.5)	41.5 (22.5)	0.23
Praying or hoping, mean (SD)	29.0 (14.3)	31.3 (17.6)	30.3 (16.2)	0.80
Catastrophizing, mean (SD)	26.1 (16.5)	41.5 (17.4)	35.3 (18.5)	0.0068
Increasing activity level, mean (SD)	48.2 (18.6)	38.1 (18.6)	42.1 (19)	0.049
Increasing pain behaviors, mean (SD)	44.9 (18.7)	47.3 (14.4)	46.3 (16)	0.73
Control over pain, mean (SD)	50.0 (31.1)	37.8 (24.3)	42.6 (27.6)	0.13
Ability to decrease pain, mean (SD)	43.3 (28.8)	38.9 (24.9)	40.6 (26.3)	0.53
**Short Form 36 (SF-36)**				
Physical functioning, mean (SD)	53.8 (23.5)	29.2 (21.3)	39 (25)	<0.001
Social functioning, mean (SD)	59.4 (27.8)	54.6 (27.2)	56.5 (22.2)	0.57
Role functioning (physical), mean (SD)	30.0 (35.9)	10.0 (22.4)	18 (29.9)	0.01
Role functioning (emotional), mean (SD)	61.7 (45.0)	35.6 (41.9)	45.9 (44.6)	0.03
Emotional well-being, mean (SD)	65.8 (16.4)	57.6 (15.0)	60.8 (15.9)	0.09
Energy/fatigue, mean (SD)	52.8 (15.3)	39.5 (13.4)	44.8 (15.5)	<0.00
Pain, mean (SD)	68.9 (19.8)	31.1 (16.5)	46.2 (25.7)	<0.00
General health, mean (SD)	48.0 (11.4)	33.2 (14.2)	39.1 (14.9)	<0.00
Health change, mean (SD)	40.0 (20.5)	24.2 (23.2)	30.5 (23.3)	<0.00

**Table 2:** Univariate analysis (ordinal logistic regression).

Variable	Motor Symptom Severity	Odds Ratio	95% Confidence Interval for OR	Standard Error
Pain presence	Mild to medium	1.32	1.31–2.41	0.56
	Medium to severe	2.95	1.65–4.26	0.67

**Table 3:** Multivariable logistic regression models.

Variables	Categories	Odds Ratio	95% CI for OR	Standard Error
Pain presence	Mild to medium	3.52	0.38–6.67	1.60
	Medium to severe	5.44	2.03–8.86	1.74
Sex	Male	4.63	1.15–18.58	3.28
Disease duration	See Table 1	1.15	0.69–1.93	0.30
Hoehn and Yahr stage	See Table 1	1.50	0.69–3.26	0.59
Pain presence (binary outcome). Adjusted for age, disease duration, and Hoehn and Yahr state. Pseudo R squared: 0.16.				

**Table 4:** Sensitivity analysis of the multivariate logistic regression model.

Variables	Categories	Odds Ratio	95% CI for OR	Standard Error
Pain intensity	Mild to medium	5.83	2.11–9.55	1.60
	Medium to severe	5.44	3.68–11.75	2.06
Sex	Male	4.63	1.15–18.58	3.28
Disease duration	See Table 1	1.15	0.69–1.93	0.30
Hoehn and Yahr stage	See Table 1	1.50	0.69–3.26	0.59
Pain intensity (ordinal outcome). Adjusted for age, disease duration, and Hoehn and Yahr state. Pseudo R-squared: 0.17.				

## Data Availability

The original contributions presented in the study are included in the article/Supplementary Materials, further inquiries can be directed to the corresponding author.

## Supplementary Materials

The following supporting information can be downloaded at https://www.mdpi.com/article/10.3390/life15020268/s1, Figure S1. Causal inference analysis of the association of pain and motor symptom severity. Red variables are confounders (variables associated with the exposure and outcome). Green variables are those that were associated with the exposure but not with the outcome. Gray variables were those that were not associated with the exposure nor outcome. The flow of the arrow indicates the explanatory pathway. We represented the variables that were considered more important according to previous clinical knowledge.
