# Supplementary material for: Association of Chronic Pain with Motor Symptom Severity in Parkinson’s Disease: An Exploratory Cross-Sectional Analysis
Source: Life (Basel). 2025 Feb 11;15(2):268. doi: 10.3390/life15020268 (PMC11856776; doi:10.3390/life15020268)
Supplement: Supplementary file 1 [file life-15-00268-s001.zip › life-3258888-supplementary.pdf]

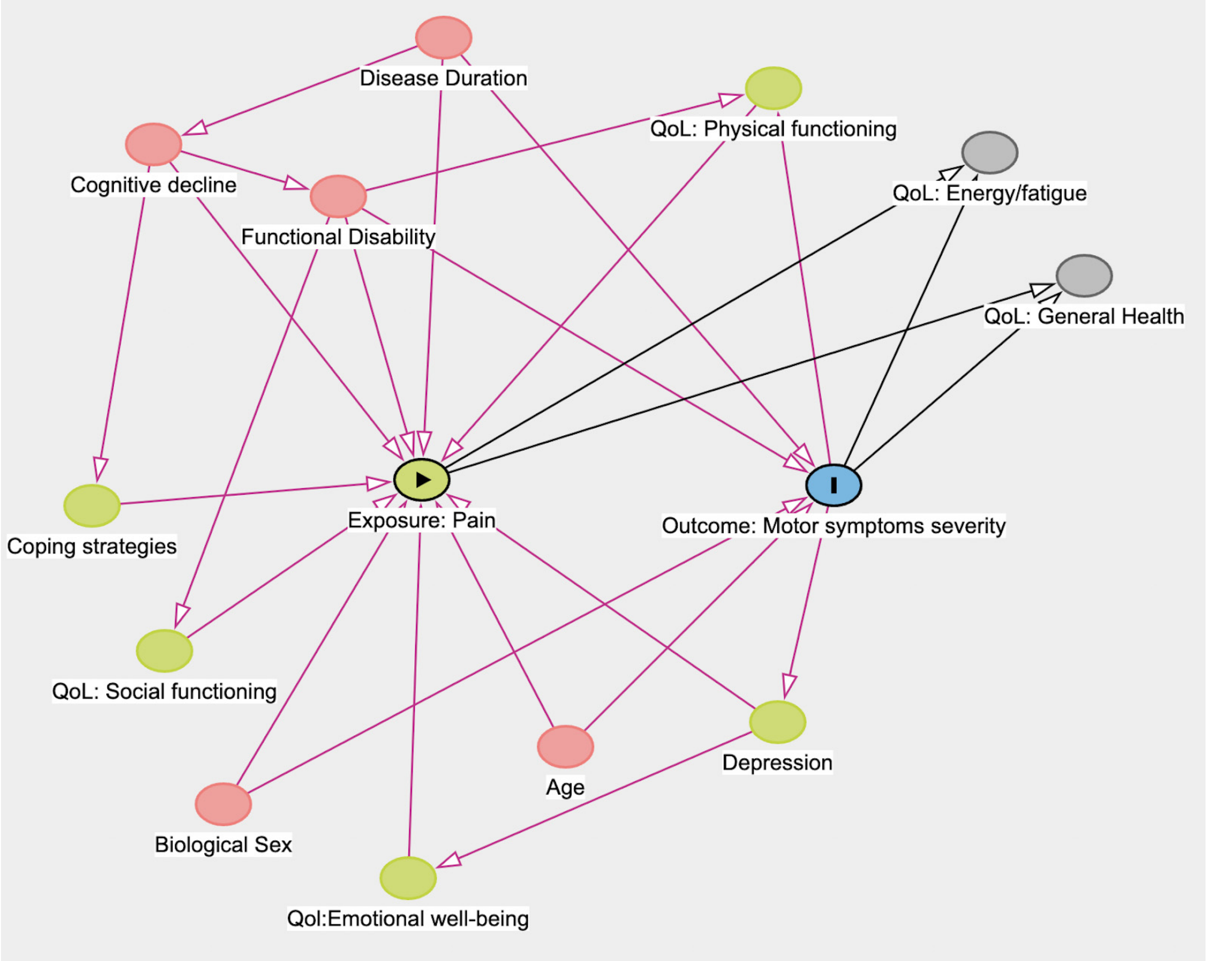

Supplementary Figure S1. Causal inference analysis of the association of pain and motor symptom severity. Red variables are confounders (variables associated with the exposure and outcome). Green variables are those that were associated with the exposure but not with the outcome. Gray variables were those that were not associated with the exposure nor outcome. The flow of the arrow indicates the explanatory pathway. We represented the variables that were considered more important according to previous clinical knowledge.
